# Supplementary material for: Activity regulates a cell type-specific mitochondrial phenotype in zebrafish lateral line hair cells
Source: eLife. 2023 Mar 13;12:e80468. doi: 10.7554/eLife.80468 (PMC10129330; doi:10.7554/eLife.80468)
Supplement: Figure 1—source data 2. [file elife-80468-fig1-data2.docx]

**Figure 1-Source Data 2: Datasets used in Figure 1**

| NM# | Dataset Name | Fish # | Genotype | Age | NM | HCs | CSCs | PSCs | Use in Figure 1 |
| --- | --- | --- | --- | --- | --- | --- | --- | --- | --- |
| NM1 | 03052021_WT_left_2 | 1 | WT | 5 dpf | SO1 | 12 | 2 | 3 | 1E-I |
| NM2 | 03052021_WT_SO1_Right | 1 | WT | 5 dpf | SO1 | 10 | 2 | 2 | 1E, 1F, 1G |
| NM3 | 03052021_WT_SO2_Right | 2 | WT | 5 dpf | SO2 | 16 | 2 | 2 | 1A-I |
| NM4 | 02102020_WT_6dpf_Right_3 | 3 | WT | 6 dpf | SO1 | 14 | 0 | 0 | 1E-I |
| NM5 | 02102020_WT_6dpf | 3 | WT | 6 dpf | SO1 | 13 | 0 | 0 | 1E, 1F, 1G |
