# Supplementary material for: Activity regulates a cell type-specific mitochondrial phenotype in zebrafish lateral line hair cells
Source: eLife. 2023 Mar 13;12:e80468. doi: 10.7554/eLife.80468 (PMC10129330; doi:10.7554/eLife.80468)
Supplement: Figure 4—source data 2. [file elife-80468-fig4-data2.docx]

**Figure 4-Source Data 2:** **Datasets used in Figure 4**

| NM# | Dataset Name | Fish # | Genotype | Age | NM | HCs | Use in Figure 4 |
| --- | --- | --- | --- | --- | --- | --- | --- |
| NM6 | 02102020_WT_3dpf_C | 4 | WT | 3 dpf | SO1 | 6 | FS1, 4A, 4A’, 4B-J, FS2 ** 2 HCs lacked ribbons |
| NM7 | 02102020_WT_3dpfA_2 | 5 | WT | 3 dpf | SO1 | 6 | FS1, 4B-J, FS2 |
