# Supplementary material for: Activity regulates a cell type-specific mitochondrial phenotype in zebrafish lateral line hair cells
Source: eLife. 2023 Mar 13;12:e80468. doi: 10.7554/eLife.80468 (PMC10129330; doi:10.7554/eLife.80468)
Supplement: Figure 5—source data 2. [file elife-80468-fig5-data2.docx]

**Figure 5-Source Data 2:** **Datasets used in Figure 5**

| NM# | Dataset Name | Fish # | Genotype | Age | NM | HCs | Use in Figure 5 |
| --- | --- | --- | --- | --- | --- | --- | --- |
| NM8 | 11262018_Opa1 | 6 | *opa1* | 5 dpf | SO1 | 5 | 5A-C |
