# Supplementary material for: Activity regulates a cell type-specific mitochondrial phenotype in zebrafish lateral line hair cells
Source: eLife. 2023 Mar 13;12:e80468. doi: 10.7554/eLife.80468 (PMC10129330; doi:10.7554/eLife.80468)
Supplement: Figure 6—source data 2. [file elife-80468-fig6-data2.docx]

**Figure 6- Source Data 2:** **Datasets used in Figure 6**

| NM# | Dataset Name | Fish # | Genotype | Age | NM | HCs | Use in Figure 6 |
| --- | --- | --- | --- | --- | --- | --- | --- |
| NM9 | 12052019_cdh23_right | 7 | *cdh23* | 5 dpf | SO1 | 5 | 6B-J, FS1 |
| NM10 | 12052019_cdh23_left | 7 | *cdh23* | 5 dpf | SO1 | 5 | 6B-J, FS1 |
| NM11 | 12192019_cdh23_IO3 | 7 | *cdh23* | 5 dpf | IO3 | 6 | 6A, 6A’, 6B-J, FS1 |
| NM12 | 12192019_cdh23_fA_SO1 | 8 | *cdh23* | 5 dpf | SO1 | 3 | 6B-J, FS1 |
