# Supplementary material for: Activity regulates a cell type-specific mitochondrial phenotype in zebrafish lateral line hair cells
Source: eLife. 2023 Mar 13;12:e80468. doi: 10.7554/eLife.80468 (PMC10129330; doi:10.7554/eLife.80468)
Supplement: Figure 7—source data 2. [file elife-80468-fig7-data2.docx]

**Figure 7-Source Data 2:** **Datasets used in Figure 7**

| NM# | Dataset Name | Fish # | Genotype | Age | NM | HCs | Use in Figure 7 |
| --- | --- | --- | --- | --- | --- | --- | --- |
| NM13 | 07292020_CaV_A_Right | 9 | *cav1.3a* | 5 dpf | SO2 | 10 | 7A, 7B-J, FS1A-F, FS1G (compared to NM3) **1 HC lacked ribbons |
| NM14 | 07292020_CaV_A_Left | 9 | *cav1.3a* | 5 dpf | SO2 | 17 | 7A’, 7B-J, FS1A-F **2 HCs lacked ribbons |
| NM15 | 07292020_CaV_fishB_right | 10 | *cav1.3a* | 5 dpf | SO2 | 13 | 7B, D-J, FS1A-F |
| NM16 | 07302020_cav_fishB_left_1 | 10 | *cav1.3a* | 5 dpf | SO2 | 8 | 7B-J, FS1A-F |
